# An imputed ancestral reference genome for the *Mycobacterium tuberculosis* complex better captures structural genomic diversity for reference-based alignment workflows

**DOI:** 10.1099/mgen.0.001165

**Published:** 2024-01-04

**Authors:** Luke B. Harrison, Vivek Kapur, Marcel A. Behr

**Affiliations:** ^1^​ Department of Medicine, McGill University, Montreal, Quebec H4A 3J1, Canada; ^2^​ Bacterial Symbionts Evolution, INRS-Centre Armand-Frappier Santé Biotechnologie, Laval, Quebec H7V 1B7, Canada; ^3^​ Department of Animal Science, The Pennsylvania State University, State College, PA 16802-3500, USA; ^4^​ McGill International TB Centre, McGill University, Montreal, Quebec H4A 3S5, Canada

**Keywords:** *Mycobacterium tuberculosis*, reference-based alignment, reference genome

## Abstract

Reference-based alignment of short-reads is a widely used technique in genomic analysis of the *Mycobacterium tuberculosis* complex (MTBC) and the choice of reference sequence impacts the interpretation of analyses. The most widely used reference genomes include the ATCC type strain (H37Rv) and the putative MTBC ancestral sequence of Comas *et al.* both of which are based on a lineage 4 sequence. As such, these reference sequences do not capture all of the structural variation known to be present in the ancestor of the MTBC. To better represent the base of the MTBC, we generated an imputed ancestral genomic sequence, termed MTBC_0_ from reference-free alignments of closed MTBC genomes. When used as a reference sequence in alignment workflows, MTBC_0_ mapped more short sequencing reads and called more pairwise SNPs relative to the Comas *et al.* sequence while exhibiting minimal impact on the overall phylogeny of MTBC. The results also show that MTBC_0_ provides greater fidelity in capturing genomic variation and allows for the inclusion of regions absent from H37Rv in standard MTBC workflows without additional steps. The use of MTBC_0_ as an ancestral reference sequence in standard workflows modestly improved read mapping, SNP calling and intuitively facilitates the study of structural variation and evolution in MTBC.

## Abbreviations

AIC, Akaike’s Information Criterion; ATCC, American Type Culture Collection; BED, Browser Extensible Data; GATK, Genome Analysis Toolkit; HAL, Hierarchical Alignment; IGV, Integrative Genomics Viewer; MRCA, Most-Recent Common Ancestor; MTBC, *Mycobacterium tuberculosis* complex; NCBI, National Center for Biotechnology Information; NGS, Next Generation Sequencing; PGAP, Prokaryotic Genome Annotation Pipeline; PHAST, Phylogenetic Analysis with Space/Time Models; RD, Region of Difference; RvD, [H37]Rv-Deletion; SNP, Single Nucleotide Polymorphism; SRA, Sequence Read Archive; TbD1, *Mycobacterium tuberculosis*-specific deletion 1.

## Data Summary

The MTBC_0_ sequence is available in the online data supplement in FASTA format at https://github.com/lukebharrison/MTBC0. Included with the MTBC_0_ sequence in the data supplement are: the reference-free alignment of MTBC closed genomes in hierarchical alignment (HAL) format, control files for cactus, annotations for H37Rv and L8, a BED file of regions excluded from SNP calls lifted over onto MTBC_0_, as well as the scripts used to call SNPs and the phylogenetic trees generated in this article. Also included is a file with per-position liftover between H37Rv and MTBC_0_. All previously published sequence data is available at the NCBI nucleotide and SRA databases, accession number for sequences used in this manuscript are available in Tables S1 and S3, available in the online version of this article.

### Impact Statement

This article describes an imputed ancestral genomic sequence (MTBC_0_) at the base of the MTBC for use as a reference sequence for *Mycobacterium tuberculosis* genomic workflows. Widely used reference sequences are limited to the structural diversity present in H37Rv, a lineage four isolate. MTBC_0_ obviates this limitation and complements pangenome approaches by incorporating the structural variation present at the base of the *Mycobacterium tuberculosis* complex (MTBC) by encompassing a wide sample of human and animal lineages including newly discovered lineages (L8, *M. orygis*). Use of MTBC_0_ enables the mapping of more reads and calling of more pair-wise SNPs and allows for the investigation of structural variation not present in the currently used reference sequences within this important group of animal and human pathogens.

## Introduction

Tuberculosis in humans and animals is caused by infection with closely related bacteria that comprise the *Mycobacterium tuberculosis* complex (MTBC). Over the past decade, studies of the phylogeny, evolution and molecular epidemiology of the MTBC have been conducted using next generation sequencing (NGS) workflows. The vast majority of NGS workflows rely on a reference-based alignment of short sequencing reads to assemble genomic sequences, call single nucleotide polymorphisms (SNPs) and investigate structural variation. Current workflows used for MTBC (e.g. MTB-seq [1]) have used either the genome of the reference strain H37Rv [2] or an estimated most-recent common ancestor of the MTBC developed by Comas *et al*. [3].

Although widely used, the choice of H37Rv is not ideal: this genome is within lineage 4 of the MTBC and thus variation called against this genome represents the sum of evolution up the tree from H37Rv to the MRCA of the MTBC and back down the lineage to the sequence in question [4]. Further, as this is a tip-to-tip comparison, the directionality of any evolutionary change is not immediately resolved. Further, regions deleted in H37Rv (referred to as Rv-Deletions [RvDs] [5]) prevent mapping of reads from those regions, even if present in the genome under investigation. This latter issue has led to the use of workarounds based on realignment of unmapped reads to an alternative reference (e.g. *Mycobacterium canettii*, for example in [6]).

Recognizing these issues, Comas *et al*. [3] proposed the use of an estimate of the MRCA of the MTBC (defined by the closed genome sampling available, using lineages L1-L6). This addressed the problem of a tip-to-tip comparison, but this sequence does not incorporate newly available genomic data and lineages (L7–9, animal lineages). Furthermore, this estimated MRCA is based on the structural variation present in the H37Rv genome and is thus unsuitable for the direct alignment of RvDs.

An alternative approach for the investigation of structural variation is to consider the MTBC pangenome. In a pangenome analysis, the totality of all genes present in the genomes under investigation, typically assembled without a reference genome, is considered [7]. This approach is well suited to investigate the complete structural variation in gene content present in a given set of genomes, including the MTBC [4]. However, its application to the MTBC, where horizontal gene transfer events are generally thought not to occur [8], and the basis of structural variation is likely limited to gene duplications, deletions and transposable elements, has lead to contradictory results, with studies concluding the MTBC has both an open and closed pangenome (reviewed in [9]). These methods can be sensitive to assembly errors, and reference-free assembly of genomes sequenced using second generation short read technology can be error prone, which may explain these contradictory results.

Given the evolutionary history of the MTBC, an ancestral genome as reference genome is attractive, as the majority of informative structural variation will likely be captured, and the sequence itself is immediately interpretable and usable in existing reference-based workflows. Thus, such an ideal reference genome for the MTBC would 1) contain the structural complement present at the root of the MTBC to maximize mapping of reads, 2) represent the ancestral state of genomic positions to polarize evolutionary events informed by the recent discovery of deeply branching MTBC lineages (e.g. L8 [10]). Here, a new estimate of the ancestral state of the genome at the root of the MTBC is derived. Its ability to better capture structural variation absent in H37Rv is demonstrated at the TbD1 and RD7/RD713/RvD4496 regions [5] and its use as a reference genome is further demonstrated with a common workflow: generation of a reference-based SNP alignment and phylogenetic tree.

## Methods

### Estimation of the ancestral genome of the MTBC

To estimate the ancestral genome of the MRCA of the MTBC, 30 closed MTBC genomes (including L1-6, L8, *M. bovis*, *M. orygis* and *M. microti*) and one *M. canettii* genome available on the NCBI GenBank database were downloaded (Table S1). Genomes were adjusted for circularity manually and softmasked for highly repetitive regions using RepeatMasker v.4.1.5 [11]. The progressiveCactus genome alignment tool was used to perform simultaneous reference-free genome alignment and estimation of ancestral genomes [12]. This algorithm requires a set phylogenetic tree, and so one was generated using a SNP alignment generated using Parsnp, executed with default parameters, using H37Rv (NC_000962.3) as a reference sequence [13]. A phylogenetic tree was estimated from this SNP alignment using RAxML v8.2.12, with the Lewis correction for ascertainment [14], and with the interrelationships of major lineages constrained to accepted relationships from recent large phylogenomic studies (e.g. [10, 15, 16]). The position of lineage 8 was collapsed into a polytomy with the two well supported major clades in the MTBC (L5, 6, 9, A1–4) and (L1–4, 7) (Fig. S1). After the initial progressiveCactus alignment and reconstruction, the haltools [12] and halPhyloP/PHAST [17] packages were used to refine ancestral state reconstructions. First, models of nucleotide evolution were fitted to the MTBC alignment using the halPhyloFit tool, and the best fit model (REV+Γ4) was chosen by minimizing the corrected Akaike’s Information Criterion (Table S2). Marginal ancestral state reconstructions at the root of the MTBC and the per site posterior probabilities were then recalculated using the best fit model and the AncestorsML tool in the haltools package, and the hierarchical alignment was updated. The NCBI prokaryotic genome annotation pipeline (PGAP, version 2023-10-03.build7061) was executed with default parameters to annotate the MTBC_0_ sequence and estimate the ancestral gene content in both the MTBC_0_ and Comas *et al.* sequences [18].

### SNP calling

A sample of 309 MTBC and one *M. canettii* genomes consisting of short reads was selected from the genomes used by Chiner-Oms *et al*. [19]: all genomes from less common lineages were included, along with sub-sampling (ten random genomes per sub-lineage) of the major MTBC lineages with extensive sampling (2,4). SRA accession numbers are provided in Table S3. Genomes identified as by Chiner-Oms as drug resistant were excluded. Raw reads were filtered and Illumina tags clipped using trimmomatic with parameters: MINLNE:20 SLIDINGWINDOW 5 : 20 TRAILING:10 [20]. Then, Kraken2 [21] was used to select only reads mapping to *Mycobacterium* and duplicate reads were removed using picard v2.23.3. Reads were aligned to a reference genome (MTBC_0_, Comas *et al.*, or H37Rv) with bwa mem [22]. GATK v4.2.2.0 was then used to call and filter SNPs and indels using GenotypeGVCF (haploid) and VariantFiltration programmes (filter parameters: QD <2.0, FS >60.0, MQRankSum <−12.5, Low40MQ, MQ <40.0, ReadPosRankSum <−8.0, DP <10). Finally, filtered SNPs and indels were further filtered to remove SNPs within repetitive and PE/PPE regions (as classified by [23]), as is standard in pipelines (e.g. MTBseq, [1]), by translating annotations using the cactus alignments in BED format from H37Rv using the halLiftover command [12]. Pairwise SNP distance between all genomes was calculated using pairsnp (https://github.com/gtonkinhill/pairsnp) for SNP calls against each reference sequence.

### Phylogenetic analysis

Filtered SNPs were used to construct a multiple sequence alignment of variable positions using samtools v1.13 [24], and a phylogenetic tree was estimated using RAxML v8.2.12, with the Lewis correction for ascertainment [14]. A rapid tree search was combined with 1000 rapid bootstrap pseudoreplicates under the GTRCAT model, followed by final optimization with the GTRGAMMA model. Phylogenies were plotted using the cophyloplot and comparePhylo tools in the phytools and APE R packages, respectively [25, 26].

### Visualization of TbD1 and RD7/RD713/RvD4496

The location of the TbD1 region (NCBI accession AJ426486.1 [5]) was identified in the MTBC_0_ reference sequence using blastn [27]. The regions of difference were visualized using the Integrative Genomics Viewer (IGV [28]) with MTBC_0_ as the reference genome, and aligned short reads from a selection of genomes in the 310 genome data set with high coverage (one per lineage other than L8 where both available genomes are included, see figures for SRA accessions). Reference annotations for H37Rv (NC_000962.3), *M. canettii* (NC_015848.1) and lineage 8 (CP048071.1) are included for context and were mapped using the cactus alignments and the halLiftover command [12]. The RD7/RD713/RvD4496 regions, as defined by Brosch *et al.*, Mostowy *et al.* and Liu *et al.* [5, 29, 30] respectively, were identified based on H37Rv annotations and visualized as above in IGV.

## Results

### MTBC_0_

**Fig. 1.**
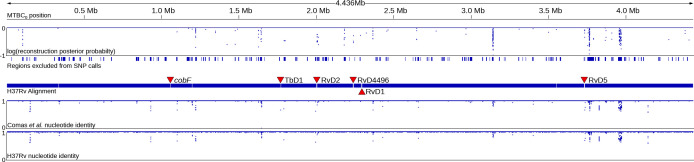
The imputed 4.436 Mb MTBC_0_ ancestral sequence, linearized, visualized in IGV and annotated. In the first track, uncertainty in ancestral state reconstruction is represented by the log posterior probabilities of the imputed nucleotide at each position, where 0 represents a probability of 1, or a certain reconstruction. In the second track, regions excluded from SNP calls in the TB SNP annotation pipeline, as published by Goig *et al*., [23] and lifted over onto MTBC_0_. Regions with increasing uncertainty in ancestral state reconstructions overlap with difficult to align regions excluded from SNP calls by Goig *et al.* The third track shows aligned regions between MTBC_0_ and H37Rv (NC_000962.3) in the hierarchical alignment. Previously published large regions (>2 kb) absent from H37Rv and present in MTBC_0_ are identified with red triangles. In the fourth and fifth tracks, the average nucleotide identity in 100 bp non-overlapping windows along the aligned blocks was calculated using an R script and the APE and SeqinR packages ([25, 34]; script available in the data supplement) and is plotted for the Comas *et al.* and H37Rv reference sequences, respectively. Divergence between MTBC_0_ and the other reference sequences is concentrated in difficult to align regions with more uncertainty in ancestral state reconstructions.

The imputed ancestral genome of the MRCA of the MTBC, MTBC_0_ is a total of 4.436 Mb in length, capturing structural variation of an additional ~24 kb relative to H37Rv (4.412 Mb). The ancestral reconstructions are generally well supported, with only 600 positions that have a posterior probability <0.95. Additionally, regions of uncertainty were concentrated in difficult-to-align regions (e.g. PE/PPE genes) which are generally excluded from SNP calling pipelines (Fig. 1). When used to align short reads from a sample of 309 MTBC genomes and one *M. canettii* genome, MTBC_0_ maps a higher proportion of filtered reads relative to H37Rv or the Comas *et al.* ancestor (summarized in Table 1, complete mapping statistics in Table S4). Using a GATK-based SNP calling pipeline with filtering for SNP quality and filtering out SNPs falling in low complexity regions as well as IS elements and PE/PPE genes, the use of MTBC_0_ as a reference calls a mean of nine and seven more pairwise SNPs relative to Comas *et al.* and H37Rv, respectively (Table 1). PGAP annotation of MTBC_0_ estimated the presence of 3961 protein coding sequences, relative to 3948 for Comas *et al.*

**Table 1.** Summary of mapping results and SNP calls for 309 short-read MTBC genomes and one *M. canettii* genome using the MTBC_0_, Comas *et al.*, and H37Rv reference sequences

Reference genome	Length (bp)	% reads mapped, median (IQR)	% reads unmapped, median (IQR)	Pairwise filtered SNPs called relative to MTBC_0_, mean (SD)	Protein coding genes (Automatic PGAP annotation)
**MTBC_0_**	4 435 783	99.7 % (0.15)	0.3 % (0.15)	–	3961
**Comas *et al.***	4 411 532	99.3 % (0.33)	0.7 % (0.33)	9 (7)	3948
**H37Rv**	4 411 532	99.3 % (0.33)	0.7 % (0.40)	7 (8)	3906 (RefSeq annotation) 3952 (PGAP re-annotation)

PGAP, Prokaryotic Genome Annotation Pipeline.

**Fig. 2.**
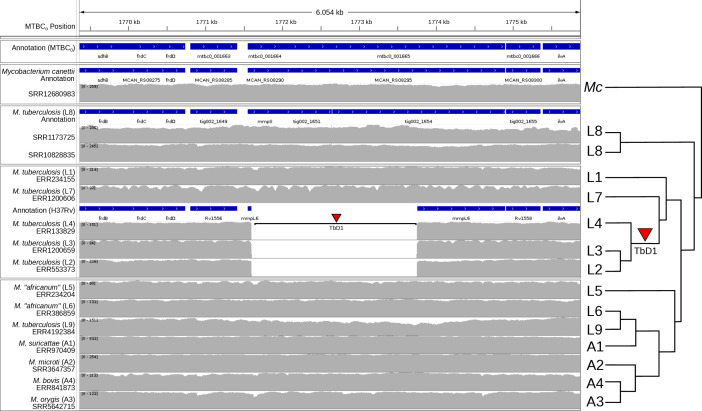
Summary of short-read alignments and log-transformed coverage for the TbD1 region relative to the MTBC_0_ reference sequence generated by IGV for a small sample of MTBC genomes and *M. canettii.* The figure is annotated on the right with a simplified phylogenetic tree topology of the MTBC estimated in Fig. S2, with arbitrary branch lengths. Note the clear presence of a large ~2.1 Kb deletion in lineages 2, 3 and 4. NCBI Prokaryotic Genome Annotation Pipeline annotation for MTBC_0_ is in the first track, and annotations for *M. canettii* (NC_015848.1), L8 (CP048071.1) and H37Rv (NC_000962.3) lifted over to MTBC_0_ are shown above their corresponding short read data sets.

**Fig. 3.**
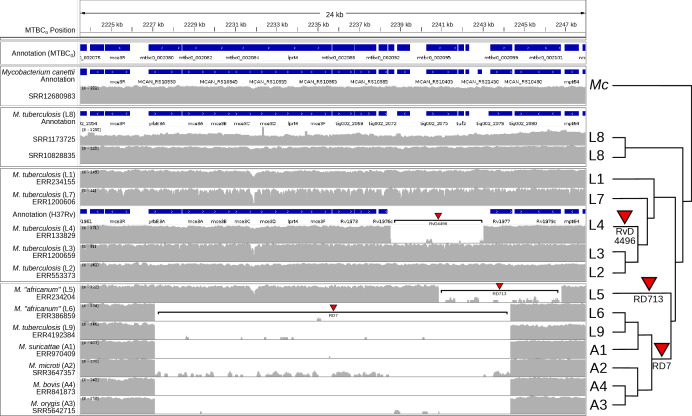
Summary of short-read alignments and log-transformed coverage for the region containing RD7, RD713, and RvD4496 relative to the MTBC_0_ reference sequence generated by IGV for a small sample of MTBC genomes and *M. canettii.* The figure is annotated on the right with a simplified phylogenetic tree topology of the MTBC estimated in Fig. S2, with arbitrary branch lengths. The RD713 described by Mostowy *et al*. [29] overlaps a deletion in H37Rv (RvD4496): using the H37Rv reference, RD713 is described as ~3.7 Kb, although a larger region is affected: ~5.9 Kb when MTBC_0_ is used as a reference. Likewise, the deleted region in RD7 is a 12.7 Kb deletion relative to H37Rv, but a 17.2 Kb deletion relative to MTBC_0_. NCBI Prokaryotic Genome Annotation Pipeline annotation for MTBC_0_ is in the first track, and annotations for *M. canettii* (NC_015848.1), L8 (CP048071.1) and H37Rv (NC_000962.3) lifted over to MTBC_0_ are shown above their corresponding short read data sets.

The TbD1 region is visualized using the MTBC_0_ reference sequence (Fig. 2). An approximately 2.1 Kb deletion is detected in lineages 2, 3 and 4. The RD7 region is similarly visualized (Fig. 3) as expected in lineages 6, 9 and A1–A4. The RD7 region also overlaps with RD713 in lineage 5 and RvD4496 in lineage 4; relative to MTBC_0_, these regions have lengths of: 4.4 Kb, 5.9 Kb and 17.2 Kb, respectively.

Phylogenetic analysis of SNP alignments generated using an identical pipeline, but with varying reference sequence shows only small differences in topology (Figs S2 and S3) within lineages, but with similar overall between lineage relationships. The topology of the phylogenetic tree is congruent with other recent analyses (e.g. [10, 15, 16]); lineage 8 is the most deeply branching lineage, and there is a deep division separating a) lineages 5, 6, 9 and the ‘animal-adapted’ lineages from b) *Mycobacterium tuberculosis sensu stricto* (lineages 1, 2, 3, 4 and 7). Major bifurcations in the tree were well supported in the bootstrap analysis for all trees.

## Discussion

The use of MTBC_0_ as a reference sequence incrementally improves mapping of reads and pairwise SNP calling relative to the Comas *et al.* sequence, albeit with only a subtle effect on a phylogeny estimated from these SNPs. The inferred phylogenetic tree is very similar (Fig. S2), and is also congruent with recent analyses, e.g. [10]. This is similar to previous work demonstrating only subtle effects on phylogeny of reference sequence choice within the MTBC [31]. Although phylogenetically informative SNPs may exist in RvDs, additional information to further address fundamental questions in the evolutionary history of the MTBC, such as whether the MRCA was human or animal-adapted, which is still an open question, will likely come from further sampling and discovery of rare and deeply branching MTBC lineages.

The use of MTBC_0_ captures structural variation, and approximately 13 more protein coding genes, not present in the Comas *et al.* sequence, and H37Rv upon which it is based. TbD1, an evolutionarily significant deletion specific to lineages 2, 3 and 4 [32] is clearly identified with MTBC_0_ used as a reference. It further demonstrates RvD4496, and clarifies the size of the overlapping RD7 and RD713 deletions, which have been underestimated relative to H37Rv/Comas *et al.* given the concomitant RvD. Previous studies using the reference sequences of H37Rv or Comas *et al.*, have relied on supplementary workflows examining unmapped reads followed by alignment against *M. canettii*. Although RvDs can be characterized in this manner, their position is then reported relative to one of the diverse *M. canettii* genomes, e.g. [30]. Other analyses may also benefit from the use of the MTBC_0_ as a reference sequence. These include the phylogenetic placement of genomes from ancient DNA analysis or from to be discovered deeply branching MTBC clades, and the intuitive analysis of regions of difference present. A final potential application is fine-grained molecular epidemiology analysis of clades distant from H37Rv that may benefit from the marginally increased SNP resolution and incorporation of SNPs present in the RvDs.

This approach has several drawbacks. The structural variation present in MTBC_0_ is based on the alignment of 30 closed genomes and is thus limited to variation present in that sample. The discovery of additional deeply branching lineages similar to what was found with L8 may reveal additional regions for future consideration and iteration. Further, as it is an estimate of the ancestral genome present at the base of the MTBC, the structural variation represented in MTBC_0_ is limited to that estimated to be present at the root of the complex. Given this, MTBC_0_ would not be ideal for studies seeking to capture and analyse the full repertoire of structural genomic diversity present in the MTBC, where a pangenome approach might be better suited. This latter concern is somewhat mitigated by the paucity of reported horizontal gene transfer events in the evolutionary history of the MTBC [8], and so MTBC_0_ will likely capture sufficient structural diversity for a large variety of use cases.

In the longer term, the continued development and refinement of long-read-based third generation sequencing technologies may enable the widespread use of reference-free workflows and pangenomics (e.g. EnteroBase [33]) that rely on *de novo* assembly. However, until the availability of long-read-based genomic data approaches that contained in the vast databases of short-read genomic sequences available and being generated for the MTBC, reference-based methods are likely to predominate. MTBC_0_ is designed to complement H37Rv and Comas *et al.*’s reference sequences, and pangenomic approaches as another tool in the toolbox, perhaps as a new ‘North star’ to facilitate genomic analyses in the MTBC, particularly for studies that need to capture the evolution of and within structural variation absent in H37Rv. Further, although not comprehensively examined here, MTBC_0_ provides a new estimate of the ancestral genomic states, both in terms of gene content and sequence. These permit intuitive interpretation of evolutionary changes and may inform estimates of ancestral phenotypic parameters in the search for the origin of the *Mycobacterium tuberculosis* complex.

## Supplementary Data

Supplementary material 1

Supplementary material 2
