# Supplementary material for: An imputed ancestral reference genome for the Mycobacterium tuberculosis complex better captures structural genomic diversity for reference-based alignment workflows
Source: Microb Genom. 2024 Jan 4;10(1):001165. doi: 10.1099/mgen.0.001165 (PMC10868604; doi:10.1099/mgen.0.001165)
Supplement: Supplementary material 1 [file mgen-10-1165-s001.pdf]

Supplementary Figure 1. Maximum likelihood phylogenetic tree estimated from the ParSNP SNP alignment derived from 30 closed MTBC genomes, with a *M. canettii* outgroup. The location of the most recent common ancestor of MTBC, and the node used for reconstruction of $MTBC_0$ is indicated as are the lineage assignments of the genomes. Note the polytomy at the base of the MTBC. Branch lengths and scale are in substitutions per site.

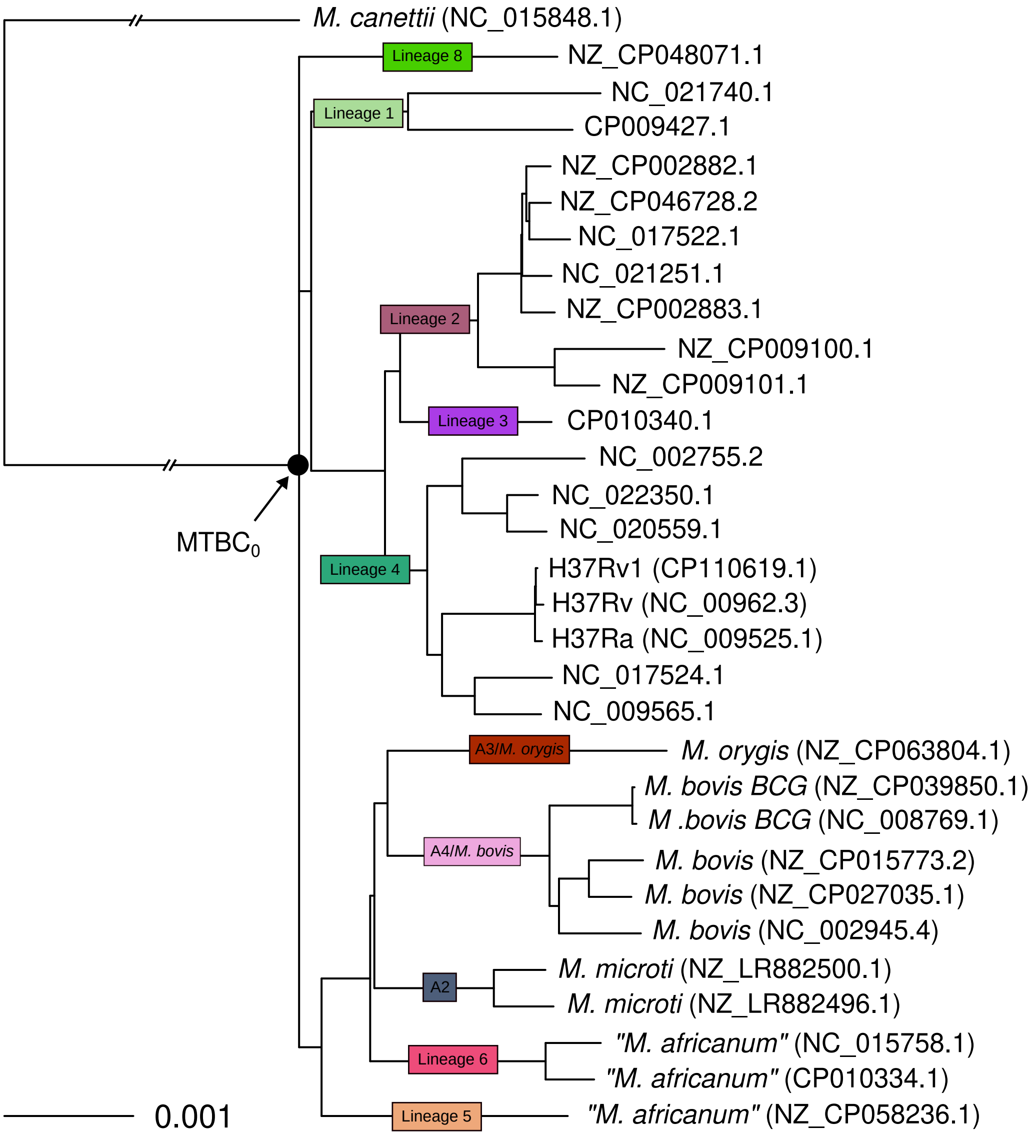

M. canettii (NC_015848.1)
Lineage 8
NZ_CP048071.1
Lineage 1
NC_021740.1
CP009427.1
NZ_CP002882.1
NZ_CP046728.2
NC_017522.1
NC_021251.1
NZ_CP002883.1
Lineage 2
NZ_CP009100.1
NZ_CP009101.1
Lineage 3
CP010340.1
NC_002755.2
NC_022350.1
NC_020559.1
MTBC0
Lineage 4
H37Rv1 (CP110619.1)
H37Rv (NC_00962.3)
H37Ra (NC_009525.1)
NC_017524.1
NC_009565.1
A3/M. orygis
M. orygis (NZ_CP063804.1)
M. bovis BCG (NZ_CP039850.1)
M .bovis BCG (NC_008769.1)
A4/M. bovis
M. bovis (NZ_CP015773.2)
M. bovis (NZ_CP027035.1)
M. bovis (NC_002945.4)
M. microti (NZ_LR882500.1)
A2
M. microti (NZ_LR882496.1)
Lineage 6
"M. africanum" (NC_015758.1)
"M. africanum" (CP010334.1)
0.001
Lineage 5
"M. africanum" (NZ_CP058236.1)

Supplementary Figure 2. Maximum likelihood phylogeny trees of the MTBC based on SNPs called from short-read sequencing data by the GATK-based pipeline, using $MTBC_0$ (left) and Comas et al., (2010) (right) reference sequences. Bootstrap values of 100% are indicated by *. Differences in phylogenetic tree topology are indicated by lines relating identical tips of the phylogeny. Branch lengths and scale are in substitutions per site.

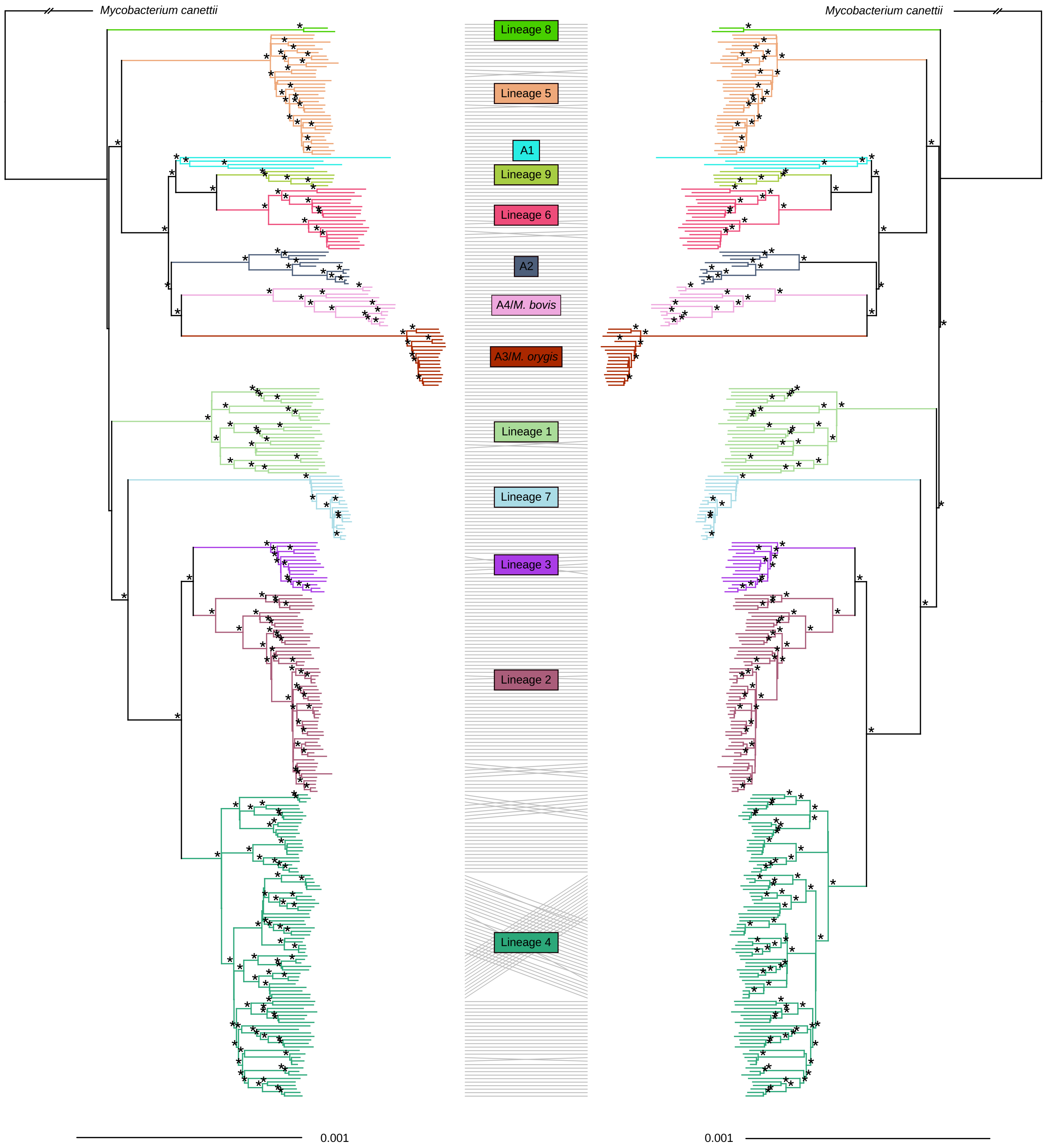

Mycobacterium canettii
Mycobacterium canettii
Lineage 8
Lineage 5
A1
Lineage 9
Lineage 6
A2
A4/M. bovis
A3/M. orygis
Lineage 1
Lineage 7
Lineage 3
Lineage 2
Lineage 4
0.001
0.001

Supplementary Figure 3. Maximum likelihood phylogeny trees of the MTBC based on SNPs called from short-read sequencing data by the GATK-based pipeline, using $MTBC_0$ (left) and H37Rv (right) reference sequences. Bootstrap values of 100% are indicated by *. Differences in phylogenetic tree topology are indicated by lines relating identical tips of the phylogeny. Branch lengths and scale are in substitutions per site.

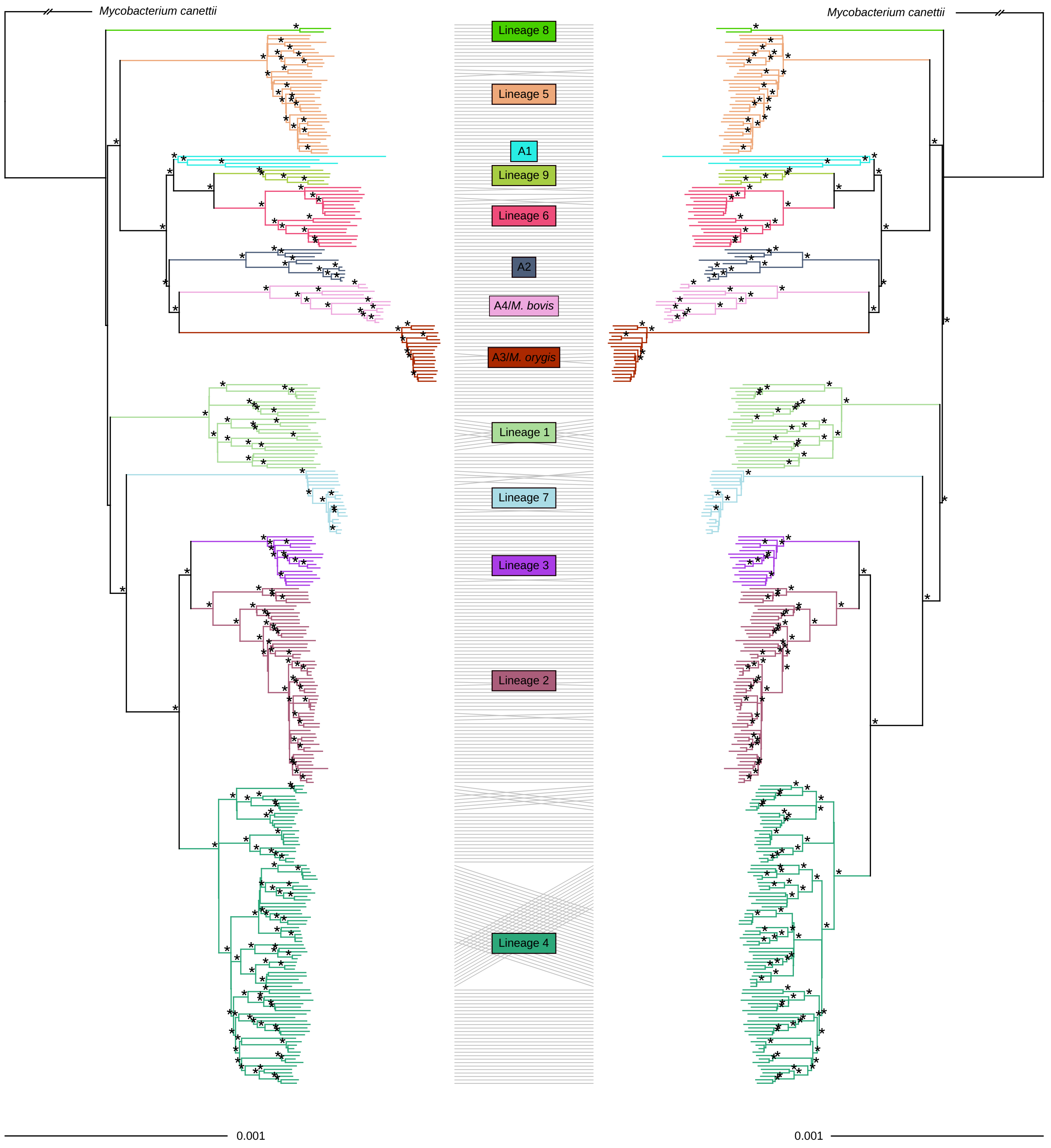

Mycobacterium canettii
Mycobacterium canettii
Lineage 8
Lineage 5
A1
Lineage 9
Lineage 6
A2
A4/M. bovis
A3/M. orygis
Lineage 1
Lineage 7
Lineage 3
Lineage 2
Lineage 4
0.001
0.001
